# Multimodal Fusion of EEG and Audio Spectrogram for Major Depressive Disorder Recognition Using Modified DenseNet121

**DOI:** 10.3390/brainsci14101018

**Published:** 2024-10-15

**Authors:** Musyyab Yousufi, Robertas Damaševičius, Rytis Maskeliūnas

**Affiliations:** Centre of Real Time Computer Systems, Kaunas University of Technology, 51368 Kaunas, Lithuania; musyyab.yousufi@ktu.edu (M.Y.);

**Keywords:** multimodal fusion, EEG, deep learning, depression, speech

## Abstract

Background/Objectives: This study investigates the classification of Major Depressive Disorder (MDD) using electroencephalography (EEG) Short-Time Fourier-Transform (STFT) spectrograms and audio Mel-spectrogram data of 52 subjects. The objective is to develop a multimodal classification model that integrates audio and EEG data to accurately identify depressive tendencies. Methods: We utilized the Multimodal open dataset for Mental Disorder Analysis (MODMA) and trained a pre-trained Densenet121 model using transfer learning. Features from both the EEG and audio modalities were extracted and concatenated before being passed through the final classification layer. Additionally, an ablation study was conducted on both datasets separately. Results: The proposed multimodal classification model demonstrated superior performance compared to existing methods, achieving an Accuracy of 97.53%, Precision of 98.20%, F1 Score of 97.76%, and Recall of 97.32%. A confusion matrix was also used to evaluate the model’s effectiveness. Conclusions: The paper presents a robust multimodal classification approach that outperforms state-of-the-art methods with potential application in clinical diagnostics for depression assessment.

## 1. Introduction

Depression and anxiety are mental disorders that affect a person’s ability to perform daily routine tasks effectively with symptoms including sleep disorder, continued sadness, bad eating habits, general cognition problems, suicidal thoughts, etc. [1,2,3]. Depression and anxiety are often comorbid conditions, with anxiety symptoms frequently preceding the onset of Major Depressive Disorder (MDD). Studies suggest that up to 60% of individuals diagnosed with MDD also experience some form of anxiety disorder. This comorbidity is so common that clinicians often find it challenging to distinguish between the two. Both disorders affect an individual’s cognitive and emotional regulation, and their co-occurrence complicates diagnosis and treatment [4,5]. According to the World Health Organization’s (WHO) report, more than 350 million people are affected by the disease worldwide [6]. Depression has emerged as the foremost cause of disability and a significant global health concern [7]. A person experiencing minor or mild depression is emotionally struggling, which can negatively influence their relationships with family, coworkers, and their ability to perform tasks effectively [8,9]. Prolonged mild depression can lead to MDD that can cause extreme mood swings, severe physiological issues, and health concerns [10]. Thus, diagnosis and prognosis of depression are crucial for it to be treated timely [11].

Diagnosing MDD involves structured clinical interviews by psychiatrists, focusing on individual moods, thoughts, behaviors, and symptoms. Also, tools like the Hamilton Depression Rating Scale (HDRS), Beck Depression Inventory (BDI), and Depression Rating Scale (DRS) are used [2]. The HDRS is a clinician administered questionnaire designed to assess depression severity, focusing on cognitive and somatic symptoms. Conversely, the BDI allows for self-assessment of mood and physical changes related to depression, offering a patient-centric view. Moreover, the American Psychiatric Association (APA) has established diagnostic criteria known as the Diagnostic and Statistical Manual of Mental Disorders (DSM-V), which is commonly used for diagnosing MDD. The DSM-V’s criteria are used globally for clinical diagnosis, research, and treatment planning for depressive disorders, ensuring consistency and standardization in the identification of mental health conditions [5].

The diagnosis of depression is inherently influenced by subjective analysis and statements from both the individual and the psychiatrist, which are subject to various factors that differ among individuals [12,13]. These factors encompass differences in symptoms, their severity, and their variability in intensity, all of which significantly affect the diagnostic process. However, despite its widespread use, the DSM-5 criteria have notable downsides. They heavily rely on subjective symptom reporting, potentially leading to variability in diagnosis among clinicians and failing to capture the full spectrum of depressive symptomatology. Neurobiological factors in MDD include dysregulation in neurotransmitters such as serotonin, norepinephrine, and dopamine, which are crucial in mood regulation. Alterations in neural connectivity, especially in areas such as the prefrontal cortex and limbic system, are also implicated in the pathophysiology of depression. The HDRS, DRS, BDI, or DSM-5 do not adequately account for the heterogeneity of depression symptoms or address potential biological markers or underlying physiological processes associated with depression [14,15]. These limitations hinder the accurate diagnosis of depression and underscore the need for further refinement of diagnostic criteria in the field.

Electroencephalography (EEG) stands as a principal technique for real-time recording, analysis, and study of brain function, owing to its ability to capture neural activity with high temporal resolution [16]. This versatility has significantly influenced the understanding and treatment of various neurological disorders as EEG’s non-invasive nature allows for monitoring brain activity in real-time and across extended periods [17,18]. The EEG device consists of one or many electrodes that are placed over the scalp at specific locations to measure electrical activity in the brain. These electrodes are positioned according to standardized protocols to ensure accurate recording of brain signals [19]. These electrodes sense the activity of different parts of the brain, capturing the changes in the voltages, between pairs of electrodes. EEG has proven to be useful in diagnosing neurological, cognitive, and psychological disorders [20]. Moreover, EEG equipment is relatively easy to maintain and less expensive compared to other modalities used for studying brain activity. This makes it particularly beneficial in settings where access to expert physicians and psychiatrists is limited [21]. EEG can be comparatively portable, adaptable to conditions and situations, and cost effective in comparison to competing imaging modalities; however, understanding an EEG recording for diagnosing a neural disorder or disease can be challenging, time-consuming, and prone to error [22]. That is attributed to the presence of noise, the intricate nature of brain electrical activity, and variances among those who operate the EEG equipment, such as specialists in the field. While the diagnosis of MDD typically requires input from psychiatrists and clinical psychologists, automated EEG analysis using ML models may offer a preliminary diagnostic tool in areas where access to mental health professionals is limited. This technology could assist in flagging potential depressive symptoms for further clinical evaluation.

Human speech is another modality that can be used for the identification of depression symptoms [13]. Speech alterations in individuals with MDD often manifest as slower speech rates, monotone prosody, and increased pauses, reflecting psychomotor retardation and cognitive slowing. These linguistic features have been used in recent studies as biomarkers for automated diagnosis of depression [23,24]. Using speech and EEG data, we suggested a framework for effortlessly diagnosing depressed patients. We have enhanced the diagnostic performance by fusing speech and EEG features at different levels and using Densenet-121 to the resulting spectra [25]. The primary findings that this study adds are as follows:

(1) A DL based classification framework for diagnosis of depression using functional brain network analysis in speech and resting-state EEG.
(2) Fusion of features extracted from the time-frequency representation of EEG and the audio spectrogram.

Further, Section 2 reviews the relevant literature. The methods are explained in Section 3. Section 4 presents experiments, experimental setups, and their results. Section 5 describes the results, and Section 6 presents a discussion. Finally, Section 7 offers the conclusions and limitations of this study.

## 2. Background

Depression is classified as a mood disorder, primarily characterized by temporary or persistent feelings of sadness, reduced pleasure, and decreased self-esteem. It is also accompanied by disorders in sleep and eating habits, difficulties in concentration, and feelings of fatigue. These symptoms may endure over an extended period, resulting in chronic and recurrent episodes that can impede an individual’s capacity to participate in daily activities. The timely identification of depression using machine learning is crucial, but it is difficult due to constraints in medical technology and skills. In the literature, numerous approaches for detecting depression are discussed, such as social media, EEG, audio and video data, and virtual reality [2,10,26].

Recently, significant advancements have been made in the field of automated early-stage depression diagnosis. These advancements leverage various data sources, including social media interactions, speech patterns, EEG readings, and other similar modalities. However, it is essential to critically evaluate these methods and their implications for diagnosis and treatment. Before proceeding, it would be beneficial to introduce some publicly available datasets related to depression disorder. Cavanagh et al. [22] described an EEG dataset with 46 MDD patients and 75 healthy controls. EEG data were collected from subjects aged 18–25 using a 64-electrode cap. To collect EEG data, the participants and physicians were given a probabilistic job to complete, which is more demanding as regards time and effort than collecting data when the subjects are at rest. Furthermore, Cai et al. [27] presented an EEG dataset of 24 MDD patients and 29 healthy controls. A 128-electrode cap was used to capture EEG data in the resting state, with eyes closed. The participants were 16–56 years old, including 33 men and 20 women. Wu et al. [28] recorded 32-channel EEGs. The MDD and healthy control dataset includes over 400 people. The average age of MDD patients was 52.85 and 54.90 years for women and men, whereas healthy patients were 49.87 and 54.59 years old. Mumtaz et al. [29] created a public database of 19-channel EEG recordings from 34 MDD patients and 30 healthy persons, with suitable gender, age, and class distribution. The data contain both eyes-open and eyes-closed resting-state data, with the MDD and healthy groups having mean ages of 40.3 ± 12.9 and 38.3 ± 15.6 years, respectively.

Mahato et al. [30] used the linear and nonlinear features for the classification of Depression. Hosseinifard et al. [31] used the EEG dataset of ninety people, half of whom were healthy controls, and extracted linear and nonlinear features and correlation dimensions for the classification. They used two algorithms, Genetic Algorithm (GA) and Support Vector Machine (SVM), for the classification of depressed and normal subjects based on EEG data, and the *Accuracy* they achieved was 88.6%. Mumtaz et al. [29] discussed the dataset and offered an ML technique for MDD classification. They used wavelet transform (WT) to obtain a feature matrix and decreased its dimension using rank-based feature selection. They used logistic regression as a classifier and their proposed technique obtained 87.5% *Accuracy*, 95% sensitivity, and 80% specificity for MDD classification. WT analysis can remove unnecessary data by compressing parameters; however, this technique is subjective, as WT studies on window functions require previous selection of frequency and time scales. Aydemir et al. [32] also developed a classification model for MDD classification using handcrafted features. They extracted features using melamine patterns and DWT, and Neighborhood Component Analysis (NCA) was used to obtain the most prominent features. They used Quadratic Support Vector Machines (SVMs) and weighted k-Nearest Neighbors (kNNs) were used for classification and achieved an *Accuracy* of 99.11% and 99.05%. As the effectiveness of the DWT technique depends on the selection of decomposition levels, and as the number of decomposition levels increases, the computational complexity of the model also escalates, potentially affecting its efficiency and applicability in clinical settings. This raises concerns about the practicality of their approach, especially in environments with limited computational resources. Furthermore, the reliance on handcrafted features may limit the model’s ability to generalize across diverse populations, suggesting the need for more automated feature extraction methods to enhance robustness and clinical utility. Erguzel et al. [33] utilized the Back Propagation Neural Network (BPNN) classification for depression. Applying BPNN to EEG data acquired from 147 individuals with MDD resulted in an *Accuracy* of 89.12%. Only six EEG channels in the delta and theta frequency ranges were used for the MDD classification. While this approach demonstrated reasonable *Accuracy*, the limitation of using a small subset of EEG channels may have reduced the model’s ability to capture the full complexity of neural activity associated with MDD. Mahato and Paul [34] used the dataset published by Mumtaz et al. [29] to create ML models for MDD classification. They used SVM, logistic regression, Naive Bayes, and decision tree classifiers. They proposed the use of different frequency bands, including alpha (8–13 Hz), alpha1 (8–10.5 Hz), alpha2 (10.5–13 Hz), beta (13–32 Hz), and delta (0.5–4 Hz). The best classification *Accuracy* was attained with alpha2 power, exceeding alpha and alpha1. Among all the classifiers, SVM performed best, reaching 88.33% *Accuracy*. Since they only used eyes-closed recordings, their ML model may benefit from more input data. Cai et al. [35] used maximum correlation and least redundancy feature selection to create a dimensionless feature space. Four classification methods were used: SVM, KNN, DT, and ANN. KNN achieved the maximum *Accuracy* of 79.27% on 92 depressed and 121 normal participants. Moreover, they observed that the absolute power of the EEG theta wave may aid in detecting depression, but the relatively low *Accuracy* suggests that more advanced methods, such as deep learning, or multimodal approaches, may be required to capture the complexity of depression symptoms more effectively. A study by Spyrou et al. [36] used an EEG dataset published by [29]. They used synchronized analysis to estimate the properties of the EEG signals. Various data classification methods, such as RF, random trees, multi-layer perceptions (MLPs), and SVM, were employed. The *Accuracy* of these classifiers ranged from 92.42% to 95.45%. Notably, the synchronization characteristics played a crucial role in the development of the classification tree, with RF achieving the highest *Accuracy* of 95.5%. While RF achieved the highest *Accuracy* the reliance on synchronization characteristics highlights a potential limitation, as this approach may overlook other significant temporal or spectral features within the EEG signals.

In addition, there has been a lot of interest in using deep learning models with EEG data [34,35,36,37] as this approach offers a new means to improve the *Accuracy* and reliability of diagnostic procedures. In prior studies, researchers manually selected features for ML to diagnose depression; however, DL can automate the process of feature selection and thus assist in the classification. Acharya et al. [37] employed a DL framework to diagnose depression and observed that the right hemisphere of the brain exhibits less distinct features compared to the left hemisphere in individuals with depression. They suggested that increasing the number of EEG electrodes could enhance model *Accuracy*. While their findings suggest hemisphere disparities, increasing electrode count in clinical settings can be difficult due to expense and complexity. Sandheep et al. [38] classified depression with Deep CNN using EEG data. They also found that the right hemisphere is better for detecting depression, and achieved a maximum *Accuracy* of 99.31%. Li et al. [39] generated the spectrograms from the EEG signals to train their proposed CNNs. They used 128-electrode EEG to classify mild depression and used both the temporal and the spatial data of EEG. As the use of 128 electrodes is a difficult in a normal clinical environment and costly, their proposed method needs improvements. Ay et al. [40] employed single-channel EEG recording with LSTM and outperformed competitors; however, their model was overfitting, making clinical usage unclear. Dand et al. [41] used a frequency-dependent multi-layer brain (FDMB) network and a CNN to diagnose MDD. The time–frequency characteristics were extracted from EEG signals, with each frequency band corresponding to a single layer of a multi-layer network. The proposed network utilized the frequency characteristics and channel coupling of EEG signals as an input to CNN-based architecture. They detected MDD with 97.27% *Accuracy*. Using three convolutional layers, performance variation between one or two layers in the core block was lower than 0.5%. Saeedi et al. [42] experimented with five distinct DL frameworks for the classification of healthy and MDD subjects. They used the generalized partial directed coherence (GPDC) and direct directed transfer function (dDTF) methods to analyze the association between EEG channels and determine effective brain connectivity. In addition, they used a novel method to create an image for each individual by combining sixteen connectivity methods. Based on the experimental analysis, the 1DCNN-LSTM model surpasses all other models in terms of performance and achieves an impressive *Accuracy* of 99.24%. Despite the 2DCNN-LSTM method achieving a faster system, it was found to be less efficient than the 1DCNN-LSTM. 

While the majority of researchers have focused on investigating a single modality for the diagnosis of depression, there is growing interest in utilizing multiple modalities for the classification of depression. According to Gupta et al. [43] a single-modality signal only provides partial information, while multi-modality signals may provide a more accurate model for identifying depression. Further, EEG analysis has also been used for identifying Alzheimer’s disease [44], detecting epileptic seizure [45] and implementing Brain–Computer Interfaces (BCIs) based on Motor Imagery [46].

This study provides a deep learning-based framework for diagnosing MDD using a multi-modal open dataset for mental-disorder analysis (MODMA). Audio speech and 128-channel resting-state EEG datasets are used from the MODMA dataset China [27]. Selected EEG channels are transformed to a Short-Time Fourier-Transform (STFT), and from the audio dataset, a Mel-spectrogram was obtained. Using Transfer Learning (TL) the pre-trained densenet121 model was trained on the datasets for feature extraction. A classification method based on feature fusion is proposed using modified Densenet-121, which enables automated classification between MDD and healthy controls. The proposed method is tested on a validation dataset, and the attained results on the speech and EEG spectrum prove greatly enhanced diagnostic performance in comparison to state-of-the-art methods.

## 3. Material and Methods

### 3.1. Dataset

In this research work, experiments were conducted using the MODMA dataset provided by Lanzhou University Second Hospital in Gansu, China [27]. The dataset consists of full-brain 128-electrode resting-state EEG recording, pervasive 3-electrode EEG recording, and audio data of depressed and normal subjects. The dataset also comprehends the statistically collected data according to the criteria of the Diagnostic and Statistical Manual of Mental Disorders IV (DSM-IV) and other scales. In this study, we used only two modalities: (i) 128-electrode full-brain resting-state EEG data; and (ii) audio data. Both data are defined below. 

**Figure 1:**
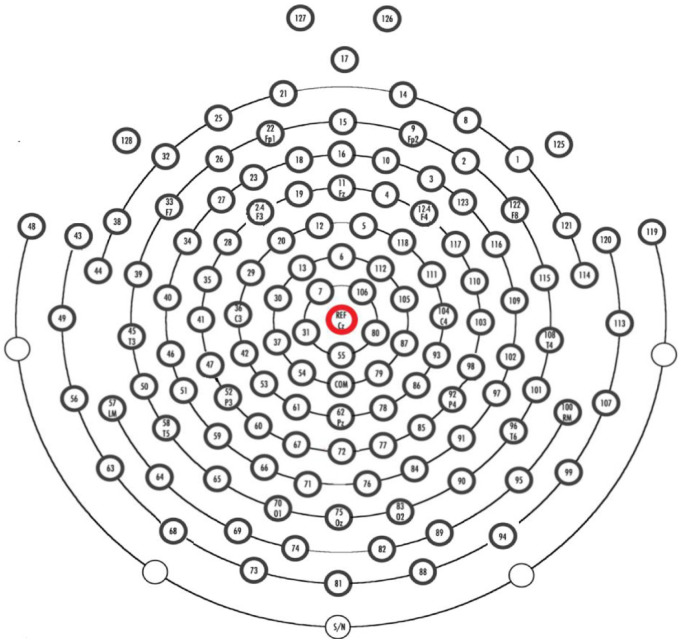
High-density 128-electrode HydroCel Geodesic Sensor net (the reference electrode is shown in red) [27].

The EEG equipment used in the study was the HydroCel Geodesic Sensor Net (HCGSN). The electrodes were Ag/AgCl, and the EEG data were collected using Net Station acquisition software version 4.5.4. The impedance of the electrodes was maintained below 50 kΩ. Full-brain 128-electrode EEG data consist of a total of 53 participants, out of which 24 are MDD patients, with 13 males and 11 females aged 16 to 56 years. The control group consists of 20 males and 9 females of ages 18–55. The EEG data were recorded for 5 min; the participants were instructed to close their eyes, be awake, and not move. The sampling frequency for recording was 250 Hz and all the electrodes were referenced to the Cz-channel as shown in Figure 1. The impedance of each electrode was kept below 50 k Ohm to ensure good contact. 

In the experiment for the audio data, 52 participants were recorded, out of which 23 were diagnosed with MDD. Out of the MDD diagnosed patients, 16 were males and 7 were females aged 16–56 years. The normal controls were 20 males and 9 females with ages of 18–55 years. The recorded audio is in the Chinese language and the experiment was conducted in the presence of psychiatrists. The experimental tasks conducted in the audio study involved participants engaging in several activities, including reading, picture description, and a question–answer session, to capture natural speech and cognitive responses. For the reading task, the participants were asked to read the short story *The North Wind and the Sun* in the Chinese language as a standard text used for acoustic analysis; also, three sets of words categorized by emotional valence (positive, neutral, and negative) from a Chinese affective word corpus were used. The picture description task involved four images: three facial expressions from the Chinese Facial Affective Picture System (CFAPS) depicting positive, neutral, and negative emotions, and one image from the Thematic Apperception Test (TAT). During the Q&A session, the participants responded to 18 interview questions adapted from the DSM-IV, covering topics ranging from positive experiences, e.g., “Describe your ideal vacation.” to emotionally neutral topics, e.g., “Describe one of your friends.”, and negative experiences, e.g., “What makes you feel hopeless?”. The entire experiment lasted approximately 25 min for each participant, including the time for all three tasks.

### 3.2. Data Processing

In this study, EEG and audio datasets were used for the classification of MDD and healthy subjects. The EEG and audio signals obtained from the source dataset were not in the T-F-Spectrogram format. For that reason, EEG data were transformed to Short-Time Fourier-Transform (STFT) and the audio data were transformed to the Mel-spectrogram. To obtain noise-free EEG signals, two primary preprocessing techniques were applied. Firstly, a notch filter centered at 50 Hz was applied to eliminate power line interference. Second, a band-pass filter with a low cutoff frequency of 0.4 Hz and high cutoff frequency of 45 Hz was used to select the specific band of EEG frequencies (awake) and to remove low-frequency and high-frequency noise, such as muscle activity. To compensate for slow baseline drifts, a sliding-window baseline correction method is utilized, where the local mean of the signal was calculated and subtracted within a moving window. No further artifact removal methods, such as Independent Component Analysis (ICA), etc., were used in the preprocessing steps. ICA is a widely recognized method for removing artifacts such as eye blinks and muscle noise in EEG data [47]. 

EEG signals are considered time-varying and demonstrate dynamic changes in frequency components. The STFT permits researchers to decompose the EEG signal into its frequency components, as there is variation over time. Despite presenting the complete signal in the frequency domain, it provides the time-dependent spectrogram, allowing us to analyze how the frequency components vary across different segments of EEG data. As the EEG signals exhibit a non-stationary and transient behavior such as outliers, measurement errors, and unusual noise, STFT appears useful in analyzing transient events. By converting EEG data to STFT, more detailed information about the temporal dynamics and frequency characteristics of neural activity can be extracted, aiding in the interpretation and analysis of the EEG signals. The mathematical definition of STFT can be provide as follows:

(1) $$X\left(M,w\right)=\sum_{s=-\infty }^{\infty }x\left(n\right)\omega \left(n-m\right)\exp^{-\iota \omega n},$$

where X(M,w) is the STFT of the signal at time index m and frequency index ω. x(n) is the input discrete signal. ω(n) is the window function. ι is the imaginary unit. The remaining STFT coefficients are as follows:

(2) $$S\left(r,w\right)={\left|S\left(r,w\right)\right|}_{\omega =2\Pi k/N},$$

where the “*N*” represents the total number of discrete frequency components. The EEG spectrogram image may be represented in time–frequency as follows:

(3) $$Spec\left(r,k\right)={\left|S\left(r,k\right)\right|}^{2},$$

For the transformation of EEG signals to STFT, the sampling rate was set to 250, the window size was set to 1024 and the hop length was set to 512. This resulted in a frequency range of 0 to 125 Hz with a frequency resolution of 0.244 Hz per bin, and a time resolution of 2.048 s per bin. Based on the findings of Hussain et al. [48], only 29 EEG channels were chosen out of 128 in this study, and the STFT spectrograms were obtained. The frontal, temporal, and parietal lobes, influence emotional and cognition processes [48,49]. The authors’ research focused on these specific regions to improve their analysis while reducing data complexity to capture and quantify important brain activity related to depression. Their research finds that the channels in these areas are more linked to MDD and cognitive function. Mel-spectrogram is the short-term power spectrum representation of an audio signal in the Mel-frequency domain and it is extensively used in audio signal processing. It can be obtained by applying the Mel-filter-bank to the squared magnitude of the Discrete Short-Time Fourier-Transform (DSTFT), as shown in the equation below:

(4) $$S_{m\;}\left[m\right]=\sum_{\omega }{\left|X\left(m,\omega \right)\right|}^{2}\cdot H_{m}\left[\omega \right],$$

where $S_{m\;}\left[m\right]$ is the Mel-spectrogram coefficient for the m-th filter at time index m, and ω represents the frequency index. $H_{m}\left[\omega \right]\;$ is the triangular Mel-filter centered at frequency index $\omega_{m}$ and $\omega_{m}$ is the center frequency index of the m-th filter.

The Mel-coefficients are treated as features, having essential information about the spectral content of the audio signal. The use of the Mel-filter-bank results in nonlinear warping of the frequency scale, converting Hz to the Mel-scale. The warping mimics the nonlinear characteristics of human hearing, that is, it is more sensitive to changes at lower frequencies. We took into consideration spectrogram-based low-level characteristics from audio data for a depression diagnosis in this experiment since recent research has shown that spectrum features performed better for speech recognition tasks. The spectrogram was extracted from the audio signal by applying the STFT on overlapping window segments. The frequency y-axis is transformed to log scale and amplitude to the color dimension to generate the spectrogram. To create the Mel-spectrogram, we translate the y-axis onto the Mel-scale. The sampling frequency of 44 KHz was used with a depth of 24 bits and the number of Mels selected was 128, while the window size was set to 1024 and the hop length was set to 512. This resulted in a frequency range of 0 to 22.05 kHz with a frequency resolution of 43.07 Hz per bin and a time resolution of 11.61 milliseconds per bin. Librosa was used in both the STFT and Mel-spectrogram cases. Librosa is an open-source Python library used for audio data analysis and processing. It can generate Mel-spectrograms of the audio data, which represent the STFT of audio signals on the Mel-frequency scale used in this study.

The EEG-to-STFT spectrograms present a fundamental tradeoff between temporal and frequency resolution. Achieving a balance between these two aspects requires a careful selection of STFT parameters, such as window size and overlap between consecutive windows. A smaller window size improves temporal resolution but compromises frequency resolution, making it difficult to distinguish between closely spaced frequency components. Conversely, a larger window size enhances frequency resolution but reduces the ability to capture rapid temporal changes. Also, the generation of Mel-spectrograms has a trade-off between temporal and frequency resolution. Mel-spectrograms provide a perceptually meaningful frequency axis for speech analysis. However, window size, hop length, and Mel-filter-bank count can greatly affect audio signal representation. A larger number of Mel-filter-banks enhances frequency resolution, allowing for finer tone element discrimination, while a smaller window size and shorter hop length improve temporal resolution. In this study, the parameters were carefully selected to ensure that critical features relevant to detecting depression are preserved. Harmony between temporal and frequency resolution can be helpful for improved classification.

### 3.3. Proposed Multimodal Depression Diagnostic Framework

In this section, we proposed a framework based on EEG and Audio Data to diagnose depression. This proposed model utilizes audio speech and resting-state EEG data acquired from the MODMA dataset. More precisely, specific EEG channels are transformed to STFT spectrograms, whereas the audio dataset is used to generate Mel-spectrograms. TL is utilized to extract features from both the audio and EEG datasets using a pre-trained DenseNet-121 model. Once the pre-trained DenseNet-121 model is loaded, the weights of its convolutional layers are frozen. During training, the parameters of these layers remain unchanged. By freezing these layers, we are able to preserve the acquired features and prevent any modifications on them while training. A custom classification layer is added on top of the frozen convolutional base. This additional layer is capable of being trained, and its purpose is to establish a connection between the extracted features and the particular categories that are significant for the given classification task, in our case, categorizing EEG and Mel-spectrogram images. Only the parameters of the custom classification layer are updated during the training process. The frozen convolutional layers serve as unchanging feature extractors, generating significant representations of the input images. The model acquires the ability to categorize the images by utilizing these extracted characteristics, while the custom classification layer adjusts its weights through the process of backpropagation and gradient descent. Mathematically, a layer in Dense Net can be represented as follows:

(5) $$x_{l}=H_{1}\left(\left[x_{0},x_{1},\ldots ,\;x_{l-1}\right]\right),$$

where $x_{l}$ is the output of layer $l,H_{1}$ are composite function consisting of batch normalization, rectified linear unit (ReLU) activation, and convolutional operations, and $\left[x_{0},x_{1},\ldots ,\;x_{l-1}\right]\;$ denotes the concatenation of feature maps from all previous layers up to l.

For our multimodal classification, we modified the pre-trained network and replaced the last fully connected layer $f_{c}$ with a custom layer for classification with two classes, including MDD and HC. Mathematically, W is the weight matrix and b is the bias vector of this fully connected layer. The output Out can be expressed as follows:

(6) $$Out=f_{c}\left(x\right)=W\cdot x+b,$$

where x is the concatenated feature vector from the preceding layers. The TL technique was used for both the datasets to extract features separately. These features are concatenated to form a fused representation, combining the information from both modalities, and are passed through the custom fully connected layer for classification.

Data augmentation and preprocessing are essential for improving machine learning model performance and robustness. This can be achieved by cleaning, transforming, and standardizing data, which enables models to handle diverse datasets and enhance performance across various tasks. For that very reason, we applied three basic data augmentation methods on all STFT EEG spectrograms and Audio Mel-spectrograms. 

First, the input data were resized to 224 × 224 pixels; this step ensures uniformity in the input size across all images in the dataset. The resize operation can be defined as follows:

(7) Resize: (H,W,C)=(224,244,3),

where H, W and C represent the height, width, and number of channels of the input image, respectively.

Secondly, the resized images were transformed into tensors. This reshapes the image data into a numerical format so that the neural network can easily process it. This transformation typically scales the pixel values to the range [0, 1].

Normalization ensures the consistent scale of input data that can improve the convergence and generalization of the neural network. We used “mean” and “Standard Deviation” for normalization of our data. Normalization works channel wise and it can be mathematically defined as follows:

(8) TNormalize=TOrignal−MeanStandard Deviation

### 3.4. Transfer Learning

A popular deep learning technique called TL makes it possible to apply a trained model to a difficult research problem. One major advantage of using TL is that it requires less input data to achieve outstanding results. It aims to transfer information from a source domain to a targeted domain, where the source domain is a huge dataset of pre-trained models, and the targeted domain is the suggested problem with few labels. The source domain typically uses a large high-resolution image dataset with billions of images and 1000 label categories are available. Using our datasets, the proposed Densenet121 model is retrained using the TL technique. TL is mathematically defined as follows:

(9) $$Z_{s}=\left\{\left(m_{1}^{s},n_{1}^{s}\right),\ldots \left(m_{j}^{s},n_{j}^{s}\right),\ldots ,\left(m_{z}^{s},n_{z}^{s}\right)\right\},$$

where $Z_{s}$ is defined as a set of pairs $\left(m_{1}^{s},n_{1}^{s}\right),\ldots \left(m_{j}^{s},n_{j}^{s}\right),\ldots ,\left(m_{z}^{s},n_{z}^{s}\right)$. Each pair $\left(m_{j}^{s},n_{j}^{s}\right)$ represents a learning task in the source domain, where $m_{j}^{s}$ is the training size data, and, $\;n_{j}^{s}$ is the label for that data. The learning tasks in the source domain are denoted as $L_{t},\;m_{j}^{s},n_{j}^{s}\in \;\phi ,$ where ϕ represents the set of learning tasks.

(10) $$Z_{t}=\left\{\left(m_{1}^{t},n_{1}^{t}\right),\ldots \left(m_{j}^{t},n_{j}^{t}\right),\ldots ,\left(m_{z}^{t},n_{z}^{t}\right)\right\},$$

where (x,y) represents training size data, where y≪x, indicating that the target domain has fewer labeled examples compared to the source domain. $\left(m_{j}^{t},\;\mathrm{and}\;n_{j}^{t}\right)$ are the labels for training data in the source and target domains, respectively. 

The pre-trained model is trained on the target datasets according to these mathematical equations, inferring that the model’s parameters are fine-tuned to the target domain using the available labeled data.

In our proposed model, transfer-learning was used to extract prominent features from the dataset. The DenseNet-121 convolutional base was used as a fixed feature extractor. After loading the pre-trained model, the weights of the convolution layer were frozen so that the parameters of these layers were not updated during the training process. This allowed the model to extract meaningful features from EEG and Mel-spectrogram images without changing pre-trained layer weights. After that, a custom fully connected layer was added to the pre-trained model to classify EEG and Mel-spectrogram images. Only this custom classification layer’s parameters were updated during training, allowing the model to map extracted features to desired output classes while preserving DenseNet-121 knowledge. This method used the pre-trained model’s knowledge to improve training and generalization, especially with a small dataset.

## 4. Experiments

### 4.1. Experimental Setup

This study investigates the *Accuracy* of the proposed approach for MDD recognition using the MODMA dataset. The labeled data were divided randomly into two parts: 80% for training the model and 20% for validating it. This split was made to ensure that the model was trained and evaluated according to the established ratio. The learning rate selected was 0.01 using the Adamax function as an optimizer. The number of Epochs was 30 and the batch size selected for image loader was 16 during the training phase of the model. Stratified cross-validation was used to conduct the experiments. The simulations were run on Ubuntu with an 11th Gen Intel(R) Core i7-11700, a 2.50 × 16 processor, 32 GB of RAM, and a trained network using an NVIDIA GeForce RTX 3080 GPU. Python 3.10.12 was used with PyTorch and Keras 2.13.0, the tool used was Jupyter Notebook 6.4.8, which runs on Linux. 

### 4.2. Evaluation Metrics

The *F1 score*, *Recall*, *Accuracy*, and *Precision* are used as performance metrics. The following is a synopsis of the assessment criteria utilized in this study.

*Accuracy* is the ratio of correct predictions to total predictions, which reflects the *Accuracy* of the classifier in making correct predictions.

(11) $$Accuracy=\frac{TN+TP}{TN+FP+TP+FN}$$

The equation above represents an *Accuracy* equation that quantifies the ratio of correctly classified data instances to all other data instances. When dealing with an unbalanced dataset, it is important to consider that *Accuracy* may not be a suitable metric. This is because the negative and positive classes have varying numbers of data instances.

*Precision* is the ratio of expected positives that are found to be true positives.

(12) $$Precision=\frac{TP}{FP+TP}$$

The *Precision* model can be found in Equation (2). It is ideal for a good classifier to have a *Precision* of 1, indicating a high level of *Accuracy*. *Precision* becomes 1 only when the numerator and denominator are equal, or when *TP* = *TP* + *FP*. This also means that *FP* is zero. When the false positive rate increases, the *Accuracy* value decreases because the denominator becomes larger than the numerator.

*Recall* is the ratio of correctly identified true positives.

(13) $$Recall=\frac{TP}{FN+TP}$$

The *Recall* equation is presented in Equation (3), where the ideal *Recall* for a good classifier is 1 (high). *Recall* reaches a value of 1 only when the numerator and denominator are the same, such as in the case of *TP* = *TP* + *FN*. This also means that *FN* is zero. As the value of *FN* increases, the denominator surpasses the numerator, resulting in a decrease in the *Recall* value.

The *F1 score* is calculated as the harmonic mean of *Recall* and *Precision*.

(14) $$F1\;Score=2*\;\frac{Precision*\mathrm{Recall}}{Precision+Recall}$$

The equation for the *F1 score* is displayed in Equation (14). When the *Precision* and *Recall* values are both 1, the *F1 score* will also be 1. When both *Precision* and *Recall* are strong, the *F1 score* can increase. Another metric that is often preferred over *Accuracy* is the *F1 score*, which calculates the harmonic mean of *Recall* and *Precision*.

### 4.3. Proposed Multimodal DenseNet121

**Figure 2:**
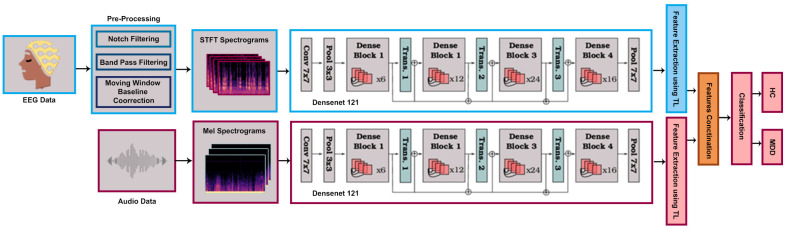
Workflow of the proposed methodology.

Our proposed methodology is established around leveraging the power of TL by incorporating a pre-trained DenseNet121 model as shown in Figure 2. The dataset, consisting of EEG and Mel-spectrogram data collected under controlled experimental conditions, undergoes preprocessing, including resizing and standardization.

**Figure 3:**
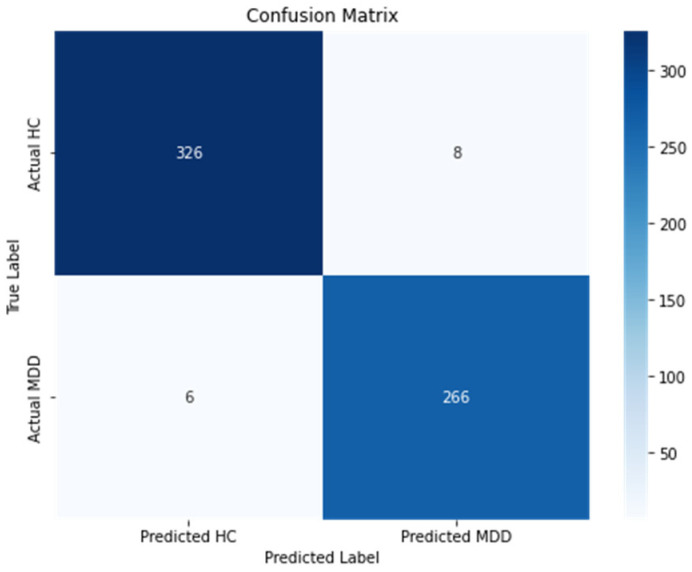
Confusion matrix of the proposed method using MODMA dataset to further analyze how each model contributed to the final fused model; experiments are performed where only EEG STFT spectrograms are used; then, only Mel-spectrograms are used for classification and the results are compared to the proposed multi-modal model.

A fundamental 80–20 train–validation split facilitates effective model learning, while data loaders efficiently handle data, leveraging batch sizes of 16. This not only optimizes memory usage but also expedites the learning process. The model includes DenseNet121, which is recognized for its dense connectivity, allowing each layer to receive input from all preceding layers. Its fully connected layer is modified and tailored to the number of classes in our datasets that are MDD and HC in both the EEG and audio datasets, then fusing the acquired features through concatenation. Training employs the “Adamax” optimizer with a learning rate of 0.001 across 30 epochs, minimizing cross-entropy loss. At the end of each epoch, the model’s performance is evaluated on the validation set using standard classification metrics, including *Accuracy*, *Precision*, recall, and *F1 score*. The confusion matrix is presented in Figure 3. The confusion matrix assigns the label “class 0” to healthy controls and “class 1” to those with MDD. 97.53%, 98.20%, 97.76%, and 97.32%.

### 4.4. Ablation Study

**Figure 4:**
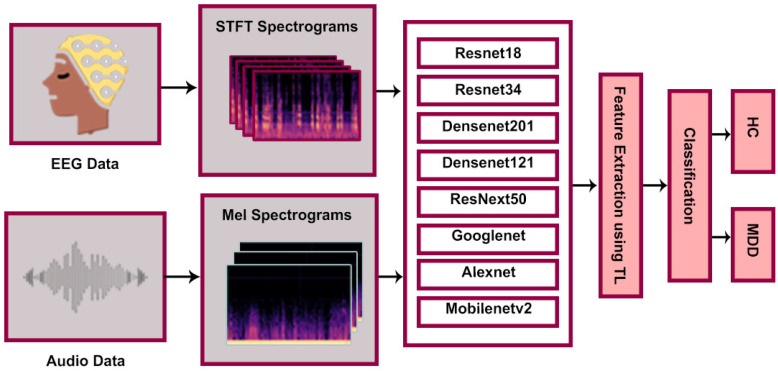
Network architectures for classification of single modality.

In this section, we performed an ablation study and used several pre-trained CNN Networks. The modified pre-trained networks for the classification of MDD and HC are shown in Figure 4.

In the first study, the EEG STFT dataset was utilized for classification, Resnet34, densenet121, ResNext50, GoogleNet, and MobileNetv2 were modified and used for classification. In this study, the data were split into 8:2 for training and validation, respectively. The data loader technique was utilized to load the spectrogram in batches, with a batch size of 32. “Admax” was used as an optimizer with a learning rate of 0.001 and the number of epochs was 30 for every pre-trained network.

In the second study, the audio Mel-spectrogram dataset was utilized for classification. Modified Resnet34, Densenet121, ResNext50, GoogleNet, and MobileNetv2 were used for classification. The data were split into 8:2 for training and validation, respectively, and the data loader technique was utilized to load the spectrogram in batches with a batch size of 32 instances. “Admax” was used as an optimizer with a learning rate of 0.001 and the number of epochs was 30 for every pre-trained network.

## 5. Results

We used multiple pre-trained networks and performed the experiments in three stages. In the first part of the experiment, we used pre-trained neural networks such as Resnet18, Resnet34, Densenet201, Densenet121, ResNext50, Google Net, AlexNet, and Mobilenetv2. The network architecture is depicted in Figure 3. The networks were trained using EEG STFT spectrograms for classification. TL was employed for each model, which improved the model’s capacity to capture complex features, particularly those that were relevant to the given scenario (in our case, to classify MDD and healthy controls). Subsequently, the model was subjected to the training, using the training dataset, and its performance was evaluated by testing it on the validation dataset. The model’s classification performances are presented in Table 1. From the results of Table 1, it can be noted that DesneNet121 outperformed the pre-trained Resnet18, Resnet34, ResNext50, Google Net, Alex Net, and MobileNetv2. For instance, the DesneNet121 model outpaced the other models in terms of *Accuracy* (97.01%), *Precision* (97.08%), *Recall* (97.08%), and F1 (97.08%) score.

For the second part of the experiment, we utilized audio Mel-spectrograms to classify participants as either MDD or normal individuals. In this case, multiple pre-trained networks were utilized, employing the TL for classification. Again, the pre-trained models underwent training using the training dataset and were subsequently validated using the validation dataset. The findings are presented in Table 2. It can be observed that Densenet-201 and Densenet-121 outperformed Resnet18, Resnet34, ResNext50, Google Net, Alex Net, and Mobilenetv2 in terms of *Accuracy*, *Precision*, recall, and *F1 score*. Densenet-121 achieved an *Accuracy* of 97.01%, *Precision* of 96.45%, *Recall* of 97.14%, and *F1 score* of 96.80%. From the results of both ablation studies, it is clear that Densent-201 and Densenet-121 are good choices for multimodal classification, but Densenet-201 is more complex and requires more powerful hardware to perform a multimodal classification task.

In the third part of the experiment, the proposed Modified Densenet121 model was trained using TL. The Densenet121 model was fed simultaneously with both EEG STFT spectrograms and audio Mel-spectrograms. The feature vectors were obtained from both modalities and concatenated before forwarding them the final classification layer. Once the classification was attained, a comparison between the proposed models was made with the already published works. As a benchmark for comparison, we employed recall, *Precision*, and *F1 score* for assessment. It can be observed that the proposed model for the classification of MDD and healthy individuals outpaced the other models in terms of *Accuracy* (97.53%), *Precision* (98.20%), *Recall* (97.32%) and *F1 score* (97.76%). Furthermore, the results of the proposed model were also analyzed using the confusion matrix, presented in Figure 3. In the confusion matrix, “class 0” represents the healthy controls, whereas “class 1” shows the MDD. The model successfully attained a true positive (TP) rate of 326, correctly classifying 326 occurrences as “Healthy Controls”. Nonetheless, eight occurrences were mistakenly categorized as “Healthy Controls” when they were in fact “MDD”, resulting in a false negative (FN) rate. In addition, the model mistakenly characterized six occurrences as “MDD” instead of “Healthy Controls”, resulting in false positive (FP) predictions. In contrast, the model accurately classified 266 occurrences as “MDD”, indicating a true negative (TN) rate. It can be observed from the confusion matrix that 2.45% of normal people are incorrectly identified as having MDD, while 2.25% of MDD are incorrectly classified as belonging to the normal class, showing the better performance of the proposed model. This analysis enables us to evaluate the model’s *Accuracy* in categorizing both “Healthy Controls” and “MDD”, offering vital insights into its efficacy in our binary classification assignment. 

Also, the performance of only the EEG spectrograms and only the audio spectrograms was compared to the fused model, which integrates both EEG and speech data. The results of this comparison are summarized in Table 3. The EEG-only model achieved an *Accuracy* of 97.33%, with a *Precision* and *Recall* of 97.34% and *F1 score* of 97.33%, demonstrating strong performance based on EEG data alone. Similarly, the Mel-spectrogram-based model achieved a slightly lower performance, with an *Accuracy* of 97.01%, a *Precision* of 96.45%, and a *Recall* of 97.14%. The fused model that integrates both EEG and speech data outperformed both individual models, achieving the highest *Accuracy* of 97.53%, *Precision* of 98.20%, and *Recall* of 97.76%. This indicates that combining EEG and speech data provides a more comprehensive analysis of the individual’s cognitive and emotional states, leading to improved classification performance.

The proposed DenseNet-121 model achieved the highest *Accuracy* at 97.53% and outperformed the other models tested in this study as shown in Table 4. It also demonstrated higher *Precision* (98.20%), *Recall* (97.76%), and *F1 score* (97.32%), indicating that it not only accurately identified MDD but also balanced the detection of true positives and true negatives effectively. In comparison, EffNetV2m, achieved a slightly lower *Accuracy* (96.21%) and a significantly lower *Recall* (91.14%), suggesting it may miss more positive cases of MDD, even though its *Precision* remained relatively high (92.83%). MobileNet, while being computationally efficient, underperformed with an *Accuracy* of 83.88%, highlighting the trade-off between model simplicity and diagnostic *Accuracy*. The results of the vision transformer are close to those of DenseNet-121, achieving an *Accuracy* of 97.31%, with nearly identical F1 score (97.30%). However, DenseNet-121’s higher *Precision* suggests better differentiation between MDD and healthy controls, making it more suitable for clinical application. These findings demonstrate DenseNet-121’s feature fusion technique between EEG and speech data, making it the most reliable automated MDD diagnostic model. Although the presented model has shown better performance, the modest dataset is one of the limitations of this study. 

## 6. Discussion

This paper focuses on the classification of MDD and healthy individuals. The multimodal MODMA dataset utilized for the classification consists of audio and resting-state EEG data of MDD and healthy individuals. The raw EEG data of the selected frequency range and optimized channels responsible for depression were transformed into STFT. The selection of EEG channels in this study was primarily based on their relevance to the brain regions associated with MDD and cognitive functioning. Also, the audio data were transformed into Mel-spectrograms. The proposed method utilized a pre-trained Densenet121 model using TL for parameter fine-tuning.

While there is a huge body of research work on the diagnostic methods for MDD detection, it can be observed that most of the studies are focused on analyzing a single modality for the diagnosis of MDD. While EEG-based studies have reported high accuracies in the detection of depression, particularly focused on specific brain regions such as the frontal lobe, they often fail to capture the behavioral and speech-related symptoms, equally important in diagnosing mood disorders. Conversely, speech-based analyses offer insights into psychomotor retardation and cognitive slowing but do not provide direct access to neurophysiological data. The findings of this study indicate that combining EEG and speech data provides a more holistic view of depression symptoms, aligning with the recent literature advocating for multimodal approaches in mental health diagnostics. By capturing both the neural and behavioral components of depression, the proposed model addresses limitations observed in single-modality studies, where reliance on a single modality may lead to misclassification or reduced *Accuracy* in certain clinical settings.

Other studies have used various approaches; for instance, Li et al. [50] achieved an *Accuracy* of 81.6% using a self-attentional CNN-BLSTM model for speech emotion recognition, which highlights the limitations of speech-only approaches). Similarly, Xin Chen and Zhigeng Pan used the decision tree model, which reached an *Accuracy* of 83.4% for depression detection based on voice data of the MODMA dataset [51]. In contrast, the speech-only model in this study demonstrated a higher *Accuracy* of 97.01%, highlighting the better performance of DL methods. Meanwhile, EEG-based approaches have also shown variable results. Wang et al. [52] implemented a temporal convolution network (TCN) and achieved an *Accuracy* of 85.23% for EEG-based depression classification, which is significantly lower than the *Accuracy* achieved in this study, suggesting that the selection of optimized EEG channels can greatly enhance performance. Qayyum et al. [13] utilized a multimodal approach, combining audio spectrograms with multiple EEG frequency bands, and applying vision transformers. The authors achieved an *Accuracy*, *Precision*, recall, and *F1 score* of 97.31%, 97.21%, 97.34%, and 97.30%, respectively, demonstrating strong performance in diagnosing depression in patients at the mild stage. Their results are comparable to those of our proposed method, where the fused model attained slightly better results. Sabbir et al. [10] proposed a novel multimodal CNN that integrates audio, video, text, and EEG data to detect depression, addressing missing modality issues through a selective dropout mechanism. Their methodology incorporates attention-based feature fusion and normalization to enhance the model’s robustness in handling incomplete data. They used three datasets: Dvlog, DAIC-WOZ, and MODMA. They achieved good *Accuracy*, *Precision*, recall, and *F1 score* with the MODMA dataset, which were 95.78%, 93.45%, 95.64%, and 94.53%. Although their results are lower compared to others, their proposed method highlights the effectiveness of their integrated multimodal approach and its potential to improve MDD classification *Accuracy* even in the presence of missing data. All results are provided in Table 4 for comparison.

It is equally important to also acknowledge the limitations of this work. The primary limitation of this work is the modest sample size. Second, the speech samples for this work were collected in a controlled setting, and it is uncertain whether the same *Accuracy* could be achieved with less structured, spontaneous speech. Third, there is a limitation related to the selection of EEG channels: although 29 channels were chosen based on prior studies, exploring additional or alternative brain regions might further enhance diagnostic *Accuracy*. Lastly, further attention can also be paid to expanding the dataset to include more participants from various demographic backgrounds to improve the generalizability of the model. Furthermore, future studies may enhance model *Accuracy* by using modalities such as textual data, facial expression data derived from images or videos, and social media content to achieve improved classification outcomes. Moreover, experimenting with other deep learning architectures, such as vision transformers or attention-based models, could further refine the feature extraction process and potentially improve performance.

## 7. Conclusions

This research study is one of the efforts to propose robust and accurate DL methods for the diagnosis of MDD by utilizing the pre-trained DenseNet-121 model. The researchers conducted experiments on the MODMA dataset that included EEG and audio data from 52 participants, including both clinically diagnosed depressed patients and non-depressed control subjects. Based on the conclusions of previous studies, a specific range of EEG frequencies and specific channels responsible for depression were selected. To produce descriptive features, the raw data were transformed to STFT from EEG and Mel-Spectrogram from audio. This approach enhances the diagnostic performance by analyzing both speech and the EEG spectrum. The proposed method has shown an *Accuracy* of 97.53 percent and has outperformed the SOTA methods. Despite the limitations of this study, including the dataset’s size, this study could be useful in clinical practices for the diagnosis of MDD.

## Figures and Tables

**Table 1:** Depression diagnosis using pre-trained EEG dataset.

Model	Accuracy (%)	Precession (%)	Recall (%)	F1 Score (%)
Resnet-18	96.75	98.37	93.80	96.03
Resnet-34	96.75	95.42	96.90	96.15
Densenet201	97.40	98.41	95.35	96.85
Densenet-121	97.40	97.08	97.08	97.08
ResNext50	96.75	96.06	96.06	96.06
Googlenet	97.73	96.92	97.67	97.30
Alexnet	74.35	74.34	62.69	68.02
Mobilenetv2	95.68	95.24	94.49	94.86

**Table 2:** Depression diagnosis using pre-trained audio data.

Model	Accuracy (%)	Precession (%)	Recall (%)	F1 Score (%)
Resnet18	96.68	97.04	95.62	96.32
Resnet34	96.68	95.77	97.14	96.45
Densenet201	98.01	96.92	98.44	97.67
Densenet121	97.01	96.45	97.14	96.80
ResNext50	95.68	93.38	36.35	35.13
Googlenet	94.68	93.60	93.60	93.60
Alexnet	86.05	80.99	88.46	84.56
Mobilenetv2	88.70	87.92	89.12	88.51

**Table 3:** Comparison of single modalities to proposed multi-modal classification model.

Model	Accuracy (%)	Precision (%)	Recall (%)	F1 Score (%)
EEG Only	97.33	97.34	97.33	97.33
Speech Only	97.01	96.45	97.14	96.80
Fused (EEG + Speech)	97.53	98.20	97.76	97.32

**Table 4:** Proposed Densenet-121 and other methods for comparisons.

Approach	Accuracy (%)	Precession (%)	Recall (%)	F1 Score (%)
Densenet121 (Proposed)	97.53	98.20	97.76	97.32
Effnetv2m (Pre-trained) [13]	96.21	92.83	91.14	93.92
Mobile-Net (Pre-trained) [13]	83.88	78.81	77.94	78.07
Vision Transformers [13]	97.31	97.21	97.34	97.30
TCN [50]	86.87	90.15	83.83	90.51
Decision Tree [51]	83.4	81.9	79.00	80.5
Multimodal + SD-Norm-Att	95.78	93.45	95.64	94.53
CNN-BLSTM [52]	81.6	-	-	-

## Data Availability

In this study, we used a Multi-modal Open Dataset for Mental Disorder Analysis (MODMA) provided by Lanzhou University Second Hospital, China, available at https://modma.lzu.edu.cn/data/index/ (accessed on 4 April 2024).
